# Subacute Combined Degeneration, Pernicious Anemia and Gastric Neuroendocrine Tumor Occured Simultaneously Caused by Autoimmune Gastritis

**DOI:** 10.3389/fnins.2019.00001

**Published:** 2019-01-25

**Authors:** Nan Zhang, Rui-Hua Li, Lin Ma, Na Li, Pei-Yan Shan, Xing-Bang Wang, Ai-Fen Liu

**Affiliations:** ^1^Department of Geriatric Medicine, Qilu Hospital of Shandong University, Jinan, China; ^2^Department of Dermatology, The Affiliated Hospital of Shandong University of Traditional Chinese Medicine, Jinan, China

**Keywords:** subacute combined degeneration, vitamin B_12_ deficiency, pernicious anemia, autoimmune gastritis, gastric neuroendocrine tumors

## Abstract

Subacute combined degeneration (SCD) is a relatively rare myelopathy mainly caused by vitamin B_12_ (VitB12) deficiency. There are many causes contributing to VitB12 deficiency. Autoimmune gastritis might lead to severe VitB12 malabsorption and in its advanced stage pernicious anemia (PA) may occur. Besides, long-term hypergastrinemia arising from achlorhydria in autoimmune gastritis is associated with neuroendocrine tumors (NETs). Patients diagnosed with SCD coexistent with PA and NET are seldomly reported. We describe a 34-year-old woman with an initial complaint of progressive fatigue, weakness and numbness in her limbs and disturbed gait. Physical examination revealed appearance of anemia, ataxia, decrease of superficial and deep sense, and positive Babinski’s sign. Laboratory tests disclosed macrocytic anemia, elevated intrinsic factor antibody and spinal MRI showed extensive T2-weighted hyperintensity in the dorsal columns. A gastric polyp was revealed by gastroscopy and histology showed an NET in the background of severe atrophic gastritis. Symptoms of the patient were relieved by a multidisciplinary therapy. In patients with SCD, PA should be suspected and prompt further investigations to elucidate causes and direct treatment.

## Background

Subacute combined degeneration (SCD) is an uncommon kind of myelopathy. It is characterized by demyelination of the lateral and dorsal columns of the spinal cord. SCD mainly results from vitamin B_12_ (VitB12) deficiency from both dietary and non-dietary causes (Stabler, 2013). Among non-dietary causes, autoimmune gastritis should not be neglected. Autoimmune gastritis is a disease of chronic inflammation of the stomach characterized by the interaction of autoantibodies against parietal cells and/or intrinsic factor (Neumann et al., 2013; Green et al., 2017). VitB12 absorption might be influenced by intrinsic factor decrease, resulting in pernicious anemia (PA) in severe conditions. Because of the autoimmune mechanism, high level of gastrin in circulation can be detected in patients with autoimmune gastritis. Persistent hypergastrinemia might increase the risk of gastric neuroendocrine tumors (NETs) (Neumann et al., 2013). The presence of coexisting SCD, PA and gastric NET is rare in young patients, and they have normal diet without history of gastrointestinal surgery. We herein describe a 34-year-old female who developed SCD, PA and gastric NET which is related to autoimmune gastritis.

## Case Presentation

A 34-year-old woman was admitted to our hospital presenting an 8-year history of progressively increasing fatigue, weakness and numbness in her limbs, especially in the distal part, and unsteady gait. Although she’d been to different hospitals several times and discontinuously got oral VitB12 and blood transfusion treatments, both hematologic and neurological symptoms presented poor improvement and even deteriorated. In the previous 20 days, the patient couldn’t walk or stand up, and she also experienced palpitations and shortness of breath. She has a history of vitiligo dating back more than 5 years. Her family history and her diet were unremarkable. A general examination revealed anemic appearance: pale palpebral conjunctivas, lips and finger nails. The neurological examination showed weakness (4/5) in the upper and lower extremities, decrease of superficial and deep sense below elbows and knees and hyperactive deep tendon reflexes in the lower extremities. The patellar clonuses, ankle clonuses, Babinski’s sign, Chaddock’s sign and Hoffmann’s sign were positive on both sides. She couldn’t complete the heel-knee-tibia test very well.

Laboratory tests disclosed macrocytic anemia: RBC (1.29^∗^10ˆ12/L, reference range 3.8–5.1^∗^10ˆ12/L), HGB (54 g/L, reference range 115–150 g/L), MCV (129.6 fL, reference range 82–100 fL), MHC (42.0 pg, reference range 27–34 pg), MCHC (324.0 g/L, reference range 316–354 g/L). The blood tests also showed decreased WBC (2.03^∗^10ˆ9/L, reference range 3.5–9.5^∗^10ˆ9/L), elevated erythrocyte sedimentation rate (ESR) (20.00 mm/h, reference range 0–18 mm/h ), normal ALT, elevated AST (70 U/L, reference range 13–35 U/L), elevated total bilirubin (30.1 μmol/L, reference range 5–21 μmol/L), elevated direct bilirubin (10.2 μmol/L, reference range < 6 μmol/L), elevated indirect bilirubin (19.9 μmol/L, reference range 2–15 μmol/L) and normal Cu (1166.2 μg/L, reference range 800–1500). Other significant laboratory results revealed a remarkably reduced level of VitB12 (<50.000 pg/ml, reference range 243–894 pg/ml), normal folate (19.26 ng/ml, reference range 3.89–19.8 ng/ml), increased intrinsic factor antibody (30.2 AU/ml, reference range < 1.53 AU/ml), elevated homocysteine (Hcy) (94.7 μmol/L, reference range < 15 μmol/L) and elevated LDH (3157U/L, reference range 120–230 U/L). Analyses of amino acids and acyl carnitine of metabolic disease in blood and organic acids in urine were unremarkable.

**FIGURE 1:**
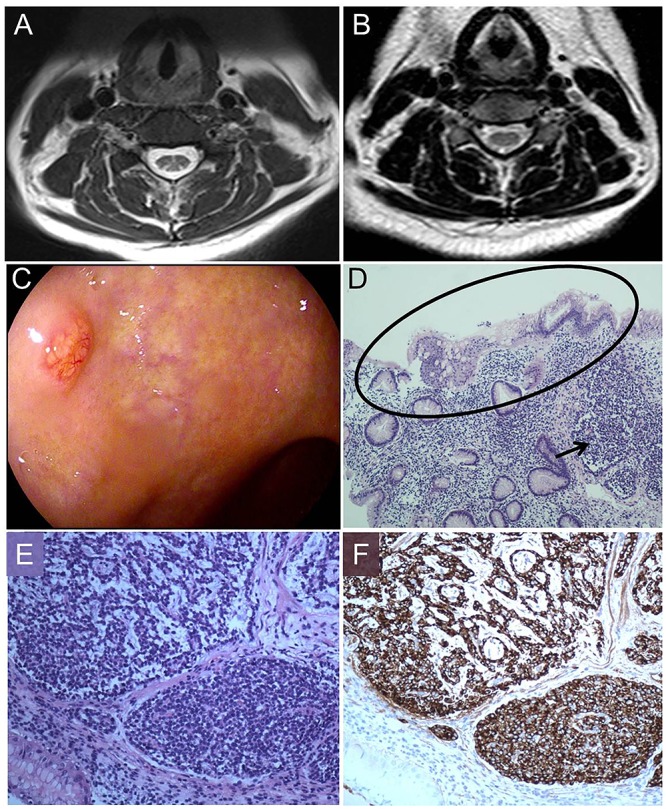
Magnetic resonance image (MRI) findings, pathologic process, morphology and immunohistochemistry. **(A)** T2–weighted image shows increased signal intensity (inverted “V” sign) in the dorsal columns of cervical cord. **(B)** After therapy, T2–weighted image shows increased signal intensity almost disappeared. **(C)** Gastroscope shows a gastric polyp. **(D)** Hematoxylin and eosin staining (100×) displays a gastric NET (arrow) in the background of reduction of intrinsic glands numbers and intestinal metaplasia (oval). **(E)** Hematoxylin and eosin staining (200×) shows nests of endocrine cells with the classical “salt and pepper” chromatin. **(F)** Staining with anti-chromogranin antibodies (CgA) (200×) depicts dark brown endocrine cells (Ki67: 2%).

The pathology of the bone marrow biopsy reported image of hyperplastic anemia. Neurogenic damage can be seen in the electroneurography and electromyography, suggesting damage of peripheral nerves in her lower limbs. The cranial magnetic resonance image (MRI) scan had no positive findings, while spinal MRI scan showed extensive T2-weighted hyperintensity in the dorsal columns from the level of C3–C6 with inverted “V” sign on axial series (Figure 1A). A gastric polyp was found by gastroscope inspection (Figure 1C), located in the mucosa and submucosa by endoscopic ultrasound (EUS) observation. The polyp proved to be NET and revealed severe chronic atrophic gastritis in pathology (Figures 1D–F).

Our patient was diagnosed with SCD, PA, gastric NET, vitiligo and hyperhomocysteinemia. A multidisciplinary therapy plan was forumlated: blood transfusions in the first week, a daily intravenous injection of 1000 μg of mecobalamine, which is a form of VitB12, for 14 days followed by 1000 μg every week through intramuscular way and endoscopic submucosal dissection (ESD) of NET.

After 14 days our patient had an easy walk and the feelings of fatigue, weakness and numbness in her limbs were mostly relieved. In the 3 months’ follow up, her anemia was corrected (RBC: 4.91^∗^10ˆ12/L, HGB: 143 g/L, MCV: 88.2 fL). AST, VitB12, bilirubin and Hcy returned to the normal levels. The T2-weighted hyperintensity of spinal MRI almost disappeared (Figure 1B).

## Discussion

In this case report, we discuss a young woman who suffered from SCD and macrocytic anemia which were explained by a remarkably reduced level of VitB12 in laboratory results. For neurologists, only testing VitB12 is not enough since VitB12 deficiency can be caused by low dietary intake, malabsorption (autoimmune gastritis, ileal disease, etc.), gastric and intestinal surgery, inherited disorders and medications (Stabler, 2013). While this patient had a balanced diet, with no family history and no gastrointestinal surgical history, malabsorption should be taken into consideration. Her elevated intrinsic factor antibody indicated the possibility of autoimmune gastritis which was confirmed by gastroscopy and there was also NET located in the mucosa and submucosa. PA was therefore the accurate clinical term for her macrocytic anemia.

Autoimmune gastritis is a chronically progressive inflammatory disease of the fundus and body of the stomach, which is characterized by the interaction of autoantibodies against parietal cells and/or intrinsic factor (Neumann et al., 2013; Green et al., 2017). On the one hand, the persistent interaction may destroy the absorption of VitB12 in the terminal ileal tract, which depends on intrinsic factors (Toh, 2014). Therefore, from a mild autoimmune gastritis to a more advanced stage, PA might occur as a consequence of severe VitB12 deficiency (Neumann et al., 2013). In the nervous system, long tracts of white matter in the dorsal and lateral columns of the spinal cord are particularly vulnerable to demyelination (known as SCD), as VitB12 is indispensable for the myelination of peripheral and central neurons (Stabler, 2013; Green, 2017; Green et al., 2017). On the other hand, oxyntic function is impaired due to the effect of parietal cell antibody. Achlorhydria continuously stimulates the gastrin-secreting cells, leading to a condition of chronic hypergastrinemia. The lasting condition might induce the proliferation of endocrine-like cells of the stomach, giving rise to a possibility of NETs (Neumann et al., 2013). A recent study showed that there was an 11-fold increased risk of developing NET in patients with PA compared with controls (Murphy et al., 2015). Although it is possible that patients with autoimmune gastritis might have SCD, PA and gastric NET at the same time, such cases are scarce in neurological clinical practice (Shinotoh et al., 1985). Through literature review, we found there was only one case report of a patient with SCD, PA, and NET simultaneously due to autoimmune gastritis (Shinotoh et al., 1985).

From our point of view, although patients with SCD concomitant with PA may not have complaint of gastrointestinal disturbance, it is important to test intrinsic factor antibody and perform a gastroscopy on SCD patients. This way, SCD patients might get an accurate diagnosis and a better management. VitB12 deficiency is the main cause of SCD and PA, so supplements of VitB12 is a key point in the management (Nagao and Hirokawa, 2017). Low-dose oral VitB12 treatment is not effective due to intrinsic factor antibody. Hence parenteral way, often intramuscular injection of VitB12, is recommended for lifelong time if the patient is diagnosed with PA (Stabler, 2013; Bizzaro and Antico, 2014). In our patient, the gastroscopy helped us diagnose autoimmune gastritis and NET which was then removed by ESD. According to NCCN guidelines, finding gastric NETs in time is crucial, as the size and numbers of NETs may change over time and managements will be quite different (Manisha et al., 2018). Once gastric NETs are removed by ESD, further surveillance is needed. It is recommended to take follow-up endoscopies every 6–12 months for the first 3 years and then at yearly intervals if there is no evidence of progression (Manisha et al., 2018).

In conclusion, although SCD is not hard to diagnose and neurologists have awareness of VitB12 testing, it is difficult to find out the reasons of VitB12 deficiency in certain circumstances. If we just give symptomatic treatment rather than seeking out the exact reasons, it might delay the effective treatment and lead to aggravation of SCD. Therefore, we suggest that SCD patients without an obvious reason for their VitB12 deficiency should be examined by intrinsic factor antibody testing and gastroscopy if necessary. When SCD is concomitant with gastrointestinal and hematic diseases as in our patient, we appeal to neurologists to work together with other specialists to give the SCD patients a comprehensive and sustained treatment. It is vital to identify the potential etiology, guide the treatment and improve the long-term prognosis.

## Concluding Remarks

When confronted with SCD patients, especially ones with VitB12 deficiency, neurologists should dig harder for possible reasons rather than just starting symptomatic treatment. It is important to test intrinsic factor antibody and carry on gastroscopy for SCD patients under certain conditions.

## Ethics Statement

Written patient consent was obtained for publication of this case report.

## Author Contributions

NZ and R-HL drafted the manuscript and literature review. LM and P-YS acquired and analyzed the data. NL interpreted the clinical data. X-BW revised and approved the final version of the manuscript. A-FL critically revised and approved the final version of the manuscript.

## Conflict of Interest Statement

The authors declare that the research was conducted in the absence of any commercial or financial relationships that could be construed as a potential conflict of interest.

## References

[B1] Bizzaro N. Antico A. (2014). Diagnosis and classification of pernicious anemia. *Autoimmun. Rev.* 13 565–568. 10.1016/j.autrev.2014.01.042 24424200

[B2] Green R. (2017). Vitamin B12 deficiency from the perspective of a practicing hematologist. *Blood* 129 2603–2611. 10.1182/blood-2016-10-569186 28360040

[B3] Green R. Allen L. H. Bjorke-Monsen A. L. Brito A. Guéant J. L. Miller J. W. (2017). Vitamin B12 deficiency. *Nat. Rev. Dis. Primers* 3:17040. 10.2147/CEG.S109123 28660890

[B4] Manisha H. S. Matthew H. K. Whitney S. G. Al B. B. Emily B. Jordan D. B. (2018). *Neuroendocrine and Adrenal Tumors, Version 1.2018 NCCN Clinical Practice Guidelines in Oncology*. Available at: https://www.nccn.org/professionals/physician_gls/default.aspx#nscl

[B5] Murphy G. Dawsey S. M. Engels E. A. Ricker W. Parsons R. Etemadi A. (2015). Cancer risk after Pernicious anemia in the US elderly population. *Clin. Gastroenterol. Hepatol.* 13 2282.e4–2289.e4. 10.1016/j.cgh.2015.05.040. 26079040 PMC4655146

[B6] Nagao T. Hirokawa M. (2017). Diagnosis and treatment of macrocytic anemias in adults. *J. Gen. Fam. Med.* 18 200–204. 10.1002/jgf2.31 29264027 PMC5689413

[B7] Neumann W. L. Coss E. Rugge M. Genta R. M. (2013). Autoimmune atrophic gastritis–pathogenesis, pathology and management. *Nat. Rev. Gastroenterol. Hepatol.* 10 529–541. 10.1038/nrgastro.2013.101 23774773

[B8] Shinotoh H. Kazahaya Y. Yamada T. Kita K. Hirayama K. (1985). Subacute combined degeneration of spinal cord. Significance of peripheral nerve involvement. *Rinsho Shinkeigaku* 25 320–326. 4017364

[B9] Stabler S. P. (2013). Clinical practice Vitamin B12 deficiency. *N. Engl. J. Med.* 368 149–160. 10.1056/NEJMcp1113996 23301732

[B10] Toh B. H. (2014). Diagnosis and classification of autoimmune gastritis. *Autoimmun. Rev.* 13 459–462. 10.1016/j.autrev.2014.01.048 24424193

